# Remission of hypothyroidism in post-bariatric surgery patients

**DOI:** 10.5339/qmj.2025.102

**Published:** 2025-12-04

**Authors:** Badurudeen Mahmood Buhary, Ali Alshehri, Muhammad Abukhater, Wafa Nawafa Mahmood, Safa Fawaza Mahmood

**Affiliations:** 1 Obesity Department, King Fahad Medical City, Riyadh, Saudi Arabia; 2 Faculty of Medicine, Kasr Al-Ainy, Cairo University, Cairo, Egypt; 3 Faculty of Medicine, David Tvildiani Medical University (AIETI), Tbilisi, Georgia *Email: safafawaza@gmail.com

**Keywords:** Hypothyroidism, bariatric surgery, obesity, thyroxine, Saudi Arabia

## Abstract

**Background:**

Hypothyroidism is commonly associated with obesity. While the effects of bariatric surgery on comorbidities such as type 2 diabetes and hypertension have been well studied, there is limited information regarding its benefits in obese patients diagnosed with and receiving treatment for hypothyroidism.

This study aimed to evaluate the effects of bariatric surgery in patients with hypothyroidism, with particular focus on changes in levothyroxine (LT4) dosage and the prevalence of hypothyroidism remission post-surgery.

**Methods:**

A retrospective study spanning seven years was conducted at the Obesity Clinic of KFMC (King Fahad Medical City—a public tertiary care center in Riyadh, Saudi Arabia), involving 163 patients with concurrent obesity and hypothyroidism who underwent bariatric surgery. Patient’s medical charts and pharmacological treatment records were reviewed. Pre- and post-operative parameters, including weight, body mass index (BMI), thyroid status, thyroid-stimulating hormone, free thyroxine, LT4 dosage, and the type of bariatric surgery performed, were recorded. Continuous variables are presented as mean±SD (standard deviation) and as percentages. Student’s t-test was used to analyze the difference between means, and data normality was assessed using the Shapiro–Wilk test.

**Results:**

Of the 163 patients, 14 (8.6%) were male and 149 (91.4%) were female, with an overall mean pre-operative BMI of 49.30 ± 9.49 kg/m^2^. Prior to surgery, the mean LT4 dose was 118.25 ± 59.39 mcg, which significantly decreased to 83.13 ± 57.39 mcg post-surgery (P < 0.001), reflecting a 30% reduction. Additionally, 24 patients (14.7%) experienced remission from hypothyroidism, whereas 8 patients (4.9%) showed an increase in thyroxine dosage.

**Conclusion:**

The study demonstrated a significant reduction in LT4 doses following bariatric surgery, suggesting that obesity may contribute to hypothyroidism. Bariatric surgery may improve thyroid function, potentially reducing the need for medication. Patients with hypothyroidism undergoing bariatric surgery should have their thyroid function closely monitored every 6–12 weeks post-operatively for 1–2 years, or until their nadir weight is reached, to allow for appropriate levothyroxine dose adjustments.

## 1. INTRODUCTION

Obesity is a global epidemic that contributes to society’s disease burden by increasing the risk of cardiovascular, metabolic, and endocrine diseases, while also decreasing the quality of life.^1,2^ Hypothyroidism is one of the most prevalent endocrine disorders and is associated with severe obesity and a higher body mass index (BMI).^1^ Patients often consider obesity a result of thyroid dysfunction; however, current understanding suggests that changes in thyroid-stimulating hormone (TSH) may instead result from obesity.^2,3^ It is recommended to measure circulating thyroid hormones in all patients with obesity to assess thyroid function.

Globally, the prevalence of hypothyroidism varies between 4% and 15%.^4–6^ Hypothyroidism is ten times more common in women than in men.^6^ In Europe, its prevalence ranges from 0.2% to 5.3%, with higher rates observed among the elderly.^7–10^ In the United States, the prevalence increased from 9.5% in 2012 to 11.7% in 2019 and is steadily increasing.^4^ In the Gulf, hypothyroidism is commonly reported and often misdiagnosed.^11^ Although no national studies have determined the population-wide prevalence of hypothyroidism in Saudi Arabia, cross-sectional studies report rates ranging from 10% to 25.5%^11–16^ and show that hypothyroidism accounts for 47% of thyroid disorders.^14^

While there have been no national studies on the prevalence of hypothyroidism in Saudi Arabia, a cross-sectional study conducted in Riyadh reported a 10% prevalence of subclinical hypothyroidism among 340 adults attending a primary care center.^11^ Globally, data on the prevalence of hypothyroidism among patients with obesity vary. The majority of the patients with obesity who do not have a diagnosed thyroid disease remain euthyroid.^17–21^ Compared to patients with normal body weight, overt and subclinical hypothyroidism are observed more frequently among patients with obesity, with estimated prevalences of 14.0% and 14.6%, respectively.^17,20–25^ An Indian study found that among the individuals with obesity, 33% had overt hypothyroidism and 11% had subclinical hypothyroidism.^26^ Obesity was more common in patients with overt hypothyroidism than in those with subclinical hypothyroidism (46% vs. 34%).^1^

Bariatric surgery is the most effective treatment option when diet, exercise, behavioral therapy, and medical management fail.^27^ In Saudi Arabia, approximately 15,000 bariatric surgeries are performed annually, and this number is expected to increase.^28^ Bariatric surgery can be restrictive, malabsorptive, or a combination of both.^28^ Laparoscopic sleeve gastrectomy (LSG) is a restrictive surgical procedure that involves removing a portion of the stomach and narrowing the remaining portion, accounting for the majority of bariatric surgeries performed. Similarly, Laparoscopic Roux-en-Y gastric bypass (LRYGB) also narrows the stomach and, in addition, bypasses a portion of the gastrointestinal tract, inducing malabsorption.^29^ The inclusion criteria set by The Saudi Arabian Society for Metabolic and Bariatric Surgery include an age of 14–65 years, a BMI of ≥35 kg/m^2^ (or ≥30 kg/m^2^ with associated comorbidities), and failure to achieve weight loss through non-surgical methods.^30^ Research indicates that bariatric surgery improves weight loss, diabetes, and cardiovascular health, with some studies also linking reductions in TSH to improvements in inflammatory markers.^31–35^ There is a paucity of data on the outcomes of bariatric surgery in hypothyroid patients. This study aimed to evaluate the effects of bariatric surgery in hypothyroid patients, with a particular focus on changes in levothyroxine (LT4) dosage and the prevalence of hypothyroid remission post-surgery.

## 2. METHODS

This retrospective observational study examined patients diagnosed with both hypothyroidism and obesity who underwent bariatric surgery at the Obesity Clinic in KFMC (King Fahad Medical City—a public tertiary center in Riyadh, Saudi Arabia) with data collected from January 2017 to October 2024. Our inclusion criteria required patients to have a BMI ≥ 35 kg/m^2^ and a diagnosis of hypothyroidism being treated with LT4. After excluding individuals who had undergone thyroidectomy or radioactive iodine ablation, 1,023 subjects were considered, of whom 190 met the inclusion criteria; 27 were further excluded due to incomplete information. Post-hoc power analysis showed that a sample size of 163 provides 93% power to detect non-inferiority with a margin of 0.0010 using a one-sided binomial test. The target significance level was 0.05, while the actual significance level achieved by the test was 0.0235. The study was approved by the Institutional Review Board (IRB) of KFMC, Saudi Arabia (IRB registration number: IRB00010471).

The following variables were collected from the medical records: age, sex, pre-operative parameters (weight, BMI, thyroid status, TSH, free thyroxine [FT4], LT4 dose, duration of hypothyroidism, glycated hemoglobin (HbA1c), and vitamin D), type of bariatric surgery performed, and post-operative parameters (nadir weight, BMI, TSH, FT4, thyroxine dose, HbA1c, and vitamin D).

Patients continued their usual treatment, and FT4, TSH, LT4 dose, and weight were recorded at each follow-up visit to assess their response to surgery. All patients were prescribed and continued on the same medication formulation, Euthyrox (MERCK Pharmaceuticals), which is provided by the Ministry of Health to all patients. Patients were instructed to take their medication early in the morning, before their predawn prayer, on an empty stomach to ensure optimal absorption without interference from food or other medications. This approach minimizes the impact of concurrent drugs that may adversely affect thyroxine absorption. Changes in weight, thyroid profile, and LT4 dosage or discontinuation were evaluated post-bariatric surgery.

Continuous variables are represented as mean ± SD (standard deviation) or as percentages. Student’s t-test was used to analyze the differences between means. The normality of the data distribution was measured using the Shapiro–Wilk test. Statistical analyses were performed using SPSS software (version 26.0; IBM Corp., Armonk, NY, USA). A p-value of < 0.05 was considered statistically significant.

## 3. RESULTS

After the initial screening process, a total of 163 patients were included in this study. Of these, 14 (8.59 %) were male and 149 (91.41%) were female, with a mean age of 45.53 ± 10.64 years (range: 17–73 years). The mean duration of diagnosed hypothyroidism was 14.15 ± 7.68 years. The distribution of bariatric surgery types among the cohort is presented in Table 1.

The effect of bariatric surgery on thyroid function was evaluated by analyzing the clinical parameters such as LT4 doses and serum levels of TSH and FT4, based on the availability of data.

The mean LT4 dose before surgery was 118.25 ± 59.39 mcg, which significantly decreased to 83.13 ± 57.39 mcg by the end of the study period, corresponding to a standard mean difference of 35.12 ± 41.35 mcg (*P* < 0.001). This represents a 30% reduction in LT4 dosage. The mean TSH before surgery was 3.61 ± 2.79 mIU/L, which decreased to 1.83 ± 1.54 mIU/L post-surgery, with a standard mean difference of 1.78 ± 2.86 mIU/L (*P* < 0.001). The mean FT4 before surgery was 12.65 ± 1.94 pmol/L and increased to 13.13 ± 1.92 pmol/L post-surgery, with a standard mean difference of −0.48 ± 2.36 pmol/L (*P* = 0.011). Thyroid peroxidase (TPO) and thyroglobulin (Tg) antibodies were tested in some patients, though not all could be assessed. Among the 15 patients tested for TPO antibodies pre-surgery, 8 were positive, with 2 turning negative post-surgery. Similarly, of 14 patients tested for Tg antibodies pre-surgery, 8 were positive, with 2 converting to negative post-surgery. Other patient characteristics are presented in Tables 2 and 3.

We also stratified the data by the type of surgery performed to determine whether any differences were present. These findings are presented in Table 4.

At the end of our study period, 24 patients (14.72%) were in remission, while 8 patients (4.90%) required an increase in thyroxine dosage. Among the 24 patients in remission, 16 had undergone LSG, 2 had LRYGB, 3 had both LSG and LRYGB, 2 had LSG and OAGB (one-anastomosis gastric bypass), and 1 had OAGB. These patients had a mean age of 40.08 ± 12.96 years (range: 17–73 years). Their mean pre-surgery weight was 132.33 ± 24.63 kg, decreasing to a post-operative nadir weight of 85.04 ± 18.22 kg. The mean pre-operative BMI was 49.78 ± 8.85, which decreased to 33.63 ± 7.43 post-operatively. Regarding thyroid function, the mean pre-operative TSH level was 3.07 ± 1.40 mIU/L, which decreased to 2.45 ± 1.03 mIU/L post-operatively (a 20.19% reduction). The mean pre-operative FT4 level was 12.60 ± 1.92 pmol/L, decreasing slightly to 11.93 ± 1.54 pmol/L post-operatively. The mean pre-operative LT4 dosage was 69.79 ± 35.33 mcg.

**Figure 1:**
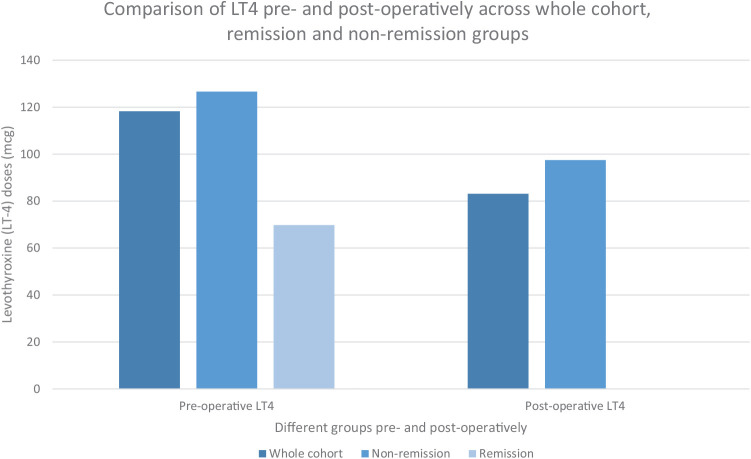
Comparison of LT4 levels across different groups: the whole cohort (all patients with hypothyroidism who underwent bariatric surgery), patients who did not achieve remission, and patients who achieved remission.

**Figure 2:**
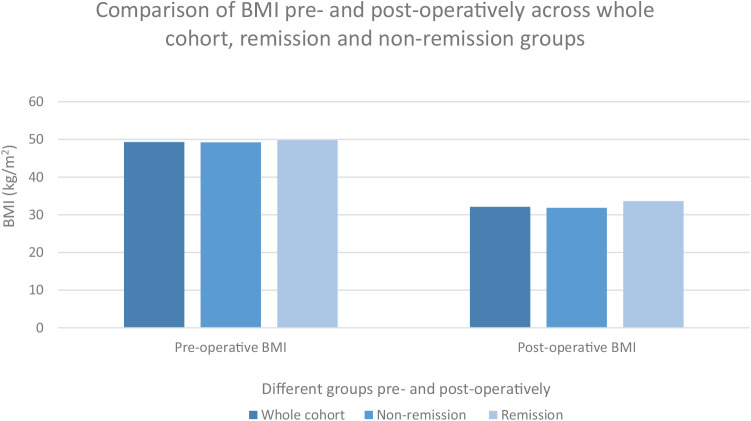
Comparison of BMI across different groups: the whole cohort (all patients with hypothyroidism who underwent bariatric surgery), patients who did not achieve remission, and patients who achieved remission.

After stratifying the data into remission and nonremission groups, we compared their mean BMI and LT4 levels before and after surgery (Figures 1 and 2). The average change in BMI was similar between the two groups. However, the mean pre-surgery LT4 dose was lower in the remission group. The pre- and post-operative parameter values for the remission patients are summarized in Table 5.

## 4. DISCUSSION

The thyroid gland plays a vital role in the human body, and thyroid disorders impose a substantial burden on the general population, affecting approximately 200 million people globally,^11^ and increasing in prevalence with rising obesity, particularly among women.^1,2^ Hypothyroidism is a common chronic condition, yet up to 60% of individuals with thyroid dysfunction remain undiagnosed due to its non-specific symptoms.^11^ If left untreated, hypothyroidism can lead to other chronic health problems, reducing both the quality of life and the economic productivity.

The prevalence of hypothyroidism is higher among individuals with obesity. A recent meta-analysis found that obesity significantly increases the risk of both overt and subclinical hypothyroidism, with a 1.86-fold higher likelihood.^24^ These conditions are bidirectionally linked, with decreased metabolic function contributing to obesity. Emerging evidence suggests that thyroid disorders may be secondary to obesity, and their etiology needs further in-depth investigation. A meta-analysis of 22 studies found that obesity was significantly associated with an increased risk of hypothyroidism (Risk ratio, RR = 1.86, 95% CI [confidence interval]: 1.63–2.11, *p* < 0.001).^24^

Obesity is characterized by a chronic low-grade inflammation that affects thyroid function through excess cytokines and inflammatory markers—such as interleukin-1 (IL-1), interleukin-6 (IL-6), and tumor necrosis factor-alpha (TNF-alpha)—produced by enlarged adipose tissues. These cytokines may inhibit the mRNA expression of the sodium/iodide symporter, which regulates iodide uptake in thyroid cells.^24^ Elevated inflammatory cytokines can cause morphological and functional changes in the thyroid by inducing vasodilation and increasing vascular permeability within the gland.^24^

A study conducted in Saudi Arabia supported these findings, demonstrating a correlation between the percentage of excess weight loss and a reduction in TSH.^6^ These results align with other studies indicating that higher body weight is associated with lower T3 and FT4 levels and higher TSH levels.^34^

Similarly, studies have shown that a reduction in TSH was correlated with decreased inflammatory markers, including IL-1, IL-6, TNF-alpha, and other cytokines, in patients with severe obesity following LSG.^34,36^

**Figure 3:**
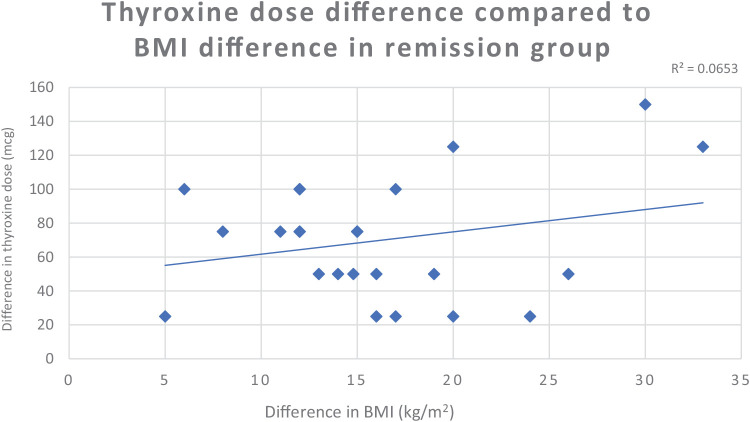
Regression analysis of changes in thyroxine dose compared to changes in BMI among patients in remission.

In our study, we observed a mild positive correlation between the change in BMI and the change in thyroxine dose among patients in remission; however, this did not reach statistical significance (*p* = 0.2280), indicating that while weight loss may contribute to remission, other factors are also likely involved (Figure 3).

Leptin levels are directly proportional to adipose tissue volume and play a key role in the relationship between obesity and hypothyroidism. Leptin contributes to chronic inflammation and can suppress sodium/iodide symporter and Tg expression, potentially leading to morphological changes in the thyroid and disrupting hormone levels in individuals with obesity.^1^ Additionally, leptin plays a role in regulating the expression of the thyrotropin-releasing hormone gene.^37,38^ These findings align with our cohort results, which showed a reduction in LT4 dosage in the majority of patients, along with remission in 14.72% of patients by the end of the study. Moreover, weight loss, which results in lower leptin levels, is also associated with a decrease in TSH levels.^2^ Our study included a higher proportion of female patients, consistent with the known gender distribution of hypothyroidism, which is approximately ten times more common in women than in men.^6^ Moreover, women are generally more likely to seek medical assistance at obesity clinics.

Research indicates that obese patients exhibit reduced expression of thyroid hormone receptor genes—particularly the TSH receptor—in both subcutaneous and visceral adipose tissues.^39,40^ Several studies have also demonstrated that bariatric surgery-induced weight loss reduces TSH levels in both euthyroid and hypothyroid patients.^25,33^ In our study, we noted a significant reduction in TSH (change in TSH (ΔTSH) = 1.78 ± 2.86, *p* < 0.0001) (Table 3).

Research indicates that ghrelin levels decrease following LSG and LRYGB, whereas they remain unchanged after laparoscopic adjustable gastric banding.^41^ The removal of the gastric fundus during surgery is associated with reduced ghrelin secretion, which may, in turn, influence TSH levels. Furthermore, the decrease in ghrelin levels is considered a contributing factor to the reduction in TSH levels, particularly as weight loss induces changes in hormone levels.^42^

Weight loss following bariatric surgery is expected to correlate with a proportional reduction in the required LT4 dose, as the recommended initial dosage for overt hypothyroidism typically ranges from 1.5 to 1.8 mcg/kg/day. Several studies have shown that thyroid function improves in hypothyroid patients with obesity following LSG and LRYGB. A study by Zendel et al. found that, out of 83 patients, 13.2% achieved remission, while the remaining patients experienced a significant decrease in LT4 dosing. The mean weekly LT4 dose decreased from 688.5 ± 40.4 mcg before surgery to 628.1 ± 45.1 mcg one year following surgery (*p* = 0.02).^42^ Another study by Alfaifi et al. found that the mean LT4 dose decreased considerably from 98.68 ± 56.18 mcg before surgery to 79.39 ± 41.49 mcg at the end of the study (*p* = 0.046).^28^ We observed similar results in our cohort, with a reduction in LT4 dosage in the majority of patients and remission achieved in 14.72% of patients by the end of the study (Table 5).

Our study also highlights the differential effects of bariatric surgery on thyroid parameters, particularly thyroxine, TSH, and FT4 levels. Among the groups, LSG showed a significant thyroxine reduction of 34.12 mcg with minimal change in FT4, while the combination of LSG and OAGB demonstrated the greatest thyroxine decrease of 50 mcg (Table 4), likely reflecting the synergistic effects of restrictive and malabsorptive mechanisms. Similarly, the combination of LSG and RYGB resulted in a significant thyroxine reduction of 47.5 mcg (Table 4), supporting the idea that combining surgical techniques may synergistically enhance metabolic and hormonal outcomes. In contrast, RYGB alone resulted in a thyroxine decrease of 27.78 mcg, which narrowly missed statistical significance (*p* = 0.051) (Table 4). FT4 changes were generally insignificant across all groups, except for LSG, which showed a modest increase of 0.52 pmol/L (Table 4). Notably, TSH levels showed more consistent and significant decreases, except in the LSG and OAGB group, where the reduction was not statistically significant, suggesting that TSH may be a more sensitive marker of thyroid function improvement following weight loss surgery. Past studies have shown mild differences in thyroid parameters among different types of bariatric surgery.^43^ Our findings suggest that combined surgeries may optimize thyroid outcomes, highlighting the need for further research into the mechanisms behind these changes and the potential advantages of integrating surgical techniques to enhance both metabolic and thyroid improvements.

A meta-analysis of 28 studies involving 1,284 patients found that bariatric surgery completely resolved subclinical hypothyroidism in 87% of cases.^44^ They also reported a statistically significant reduction in LT4 dose among patients with overt hypothyroidism following bariatric surgery (mean difference: −13.20 mcg/day, 95% CI: [−19.69, −6.71]).^44^ In contrast, all patients in our study had overt hypothyroidism, differing from previous studies.

We noted that the mean BMI percentage change for the entire cohort, the non-remission group, and the remission group was 34.89%, 35.29%, and 32.44%, respectively (Figure 2). This indicates that factors other than weight loss may contribute to remission in these patients.

The limitations of this study include its retrospective design and the higher proportion of female patients compared to males, which reflects both the greater number of female patients presenting to the clinic and the higher prevalence of hypothyroidism among females. Future prospective studies are needed to provide deeper insights into this topic.

Our study demonstrated an association between bariatric surgery and reduced LT4 dosage requirements in hypothyroid patients, suggesting that obesity may contribute to hypothyroidism. Following bariatric surgery, thyroid profiles improved significantly. The thyroid function in obesity requires careful monitoring and management. The relationship between obesity and thyroid disease is bidirectional and complex, necessitating further in-depth studies to clarify its underlying mechanisms.

## 5. CONCLUSION

The study was conducted at a single-center obesity clinic in a tertiary hospital in Saudi Arabia, which included a higher proportion of female patients. A significant reduction in LT4 doses was observed following bariatric surgery, suggesting that obesity may contribute to hypothyroidism. Notably, the average change in BMI before and after surgery was similar in both the remission and non-remission groups. Bariatric surgery may improve thyroid function, potentially reducing the need for medication. Patients with hypothyroidism undergoing bariatric surgery should be closely monitored every 6–12 weeks post-operatively for thyroid function for up to 1–2 years, or until the nadir weight is reached, to allow appropriate dose adjustments. Patients may also achieve remission and/or require frequent dose adjustments based on their weight loss; thus, close monitoring of thyroid function is essential.

## AUTHOR’S CONTRIBUTION

BMB: Contributed to idea conceptualization, patient care, data collection, and manuscript preparation. AA and MA: Contributed to patient care and data collection. WNM and SFM: Contributed to manuscript preparation and statistical analysis.

## COMPETING INTERESTS

The authors have no conflicts of interest to declare.

## Figures and Tables

**Table 1.** Distribution of patients by gender and type of bariatric surgery.

Type of bariatric surgery done	Total	Male	Female
Total number of patients	163	14	149
LSG	126	8	118
LSG+OAGB	6	0	6
LSG+RYGB	10	3	7
BPD/DS	1	0	1
RYGB	18	3	15
OAGB	2	0	2

LSG: Laparoscopic sleeve gastrectomy; OAGB: One-anastomosis gastric bypass; RYGB: Roux-en-Y gastric bypass; BPD/DS: Biliopancreatic diversion with duodenal switch.

**Table 2.** Pre- and post-operative parameter values for the whole cohort (all patients with hypothyroidism who underwent bariatric surgery).

	Mean ± SD	*N*
Pre-operative weight (kg)	125.65 ± 23.17	163
Post-operative nadir weight (kg)	81.84 ± 17.66	163
Pre-operative body mass index (kg/m^2^)	49.30 ± 9.49	163
Post-operative body mass index (kg/m^2^)	32.10 ± 7.05	163
Pre-operative thyroid-stimulating hormone (mIU/L)	3.62 ± 2.79	163
Post-operative thyroid-stimulating hormone (mIU/L)	1.83 ± 1.54	163
Pre-operative free thyroxine (pmol/L)	12.65 ± 1.94	163
Post-operative free thyroxine (pmol/L)	13.13 ± 1.92	163
Pre-operative thyroxine dose (mcg)	118.25 ± 59.39	163
Post-operative thyroxine dose (mcg)	83.13 ± 57.39	163
Pre-operative HbA1c (%)	6.42 ± 1.62	163
Post-operative HbA1c (%)	5.43 ± 0.68	163
Pre-operative vitamin D (nmol/L)	60.41 ± 24.82	162
Post-operative vitamin D (nmol/L)	83.87 ± 28.41	162

HbA1c: Hemoglobin A1c.

**Table 3.** Mean difference in parameters before and after surgery for the whole cohort (all patients with hypothyroidism who underwent bariatric surgery).

Number of patients, *n* = 163	Mean ± SD	*p*-value
Variation in weight (kg)	43.81 ± 17.18	<0.0001
Variation in body mass index (kg/m^2^)	17.19 ± 7.30	<0.0001
Variation in thyroid-stimulating hormone (μIU/mL)	1.78 ± 2.86	<0.0001
Variation in free thyroxine (ng/dL)	−0.48 ± 2.36	0.011
Variation in thyroxine dose (mcg)	35.12 ± 41.35	<0.0001
Variation in HbA1c (%)	0.99 ± 1.33	<0.0001
Variation in vitamin D (nmol/L) [*n* = 162]	−23.45 ± 32.68	<0.0001

HbA1c: Hemoglobin A1c.

**Table 4.** Mean difference in parameters before and after surgery, stratified by the type of surgery.

	Laparoscopic sleeve gastrectomy (*n* = 126)		Laparoscopic sleeve gastrectomy+one-anastomosis gastric bypass (*n* = 6)		Laparoscopic sleeve gastrectomy+Roux-en-Y gastric bypass (*n* = 10)		Roux-en-Y gastric bypass (*n* = 18)	
	Mean ± SD	*p*-value	Mean ± SD	*p*-value	Mean ± SD	*p*-value	Mean ± SD	*p*-value
Variation in weight (kg)	42.21 ± 17.05	<0.0001	48.17 ± 13.76	>0.001	54.00 ± 17.33	>0.001	44.56 ± 16.69	>0.001
Variation in body mass index (kg/m^2^)	16.64 ± 7.33	<0.0001	21.80 ± 7.34	0.001	20.40 ± 7.19	>0.001	16.67 ± 6.48	>0.001
Variation in thyroid-stimulating hormone (μIU/mL)	1.95 ± 3.11	<0.0001	0.19 ± 0.62	0.492	1.10 ± 1.46	0.041	1.66 ± 1.96	0.002
Variation in free thyroxine (ng/dL)	−0.52 ± 2.52	0.022	0.37 ± 0.95	0.387	−0.60 ± 1.08	0.114	−0.40 ± 2.27	0.458
Variation in thyroxine dose (mcg)	34.13 ± 38.80	<0.0001	50.00 ± 15.81	0.001	47.50 ± 51.98	0.018	27.78 ± 56.16	0.051
Variation in HbA1c (%)	0.93 ± 1.22	<0.0001	1.97 ± 2.81	0.147	0.97 ± 1.64	0.095	1.08 ± 1.31	0.003
Variation in vitamin D (nmol/L) [*n* = 162]	−26.77 ± 32.95	<0.0001	1.07 ± 19.85	0.900	−18.56 ± 25.04	0.044	−9.61 ± 34.50	0.254

**Table 5.** Pre- and post-operative parameter values for patients in remission.

	Mean ± SD	*n*
Pre-Op weight (kg)	132.33 ± 24.63	24
Post-Op nadir weight (kg)	85.04 ± 18.22	24
Pre-Op BMI (kg/m^2^)	49.78 ± 8.85	24
Post-Op BMI (kg/m^2^)	33.63 ± 7.43	24
Pre-Op TSH (mIU/L)	3.07 ± 1.40	24
Post-Op TSH (mIU/L)	2.45 ± 1.04	24
Pre-Op FT4 (pmol/L)	12.60 ± 1.93	24
Post-Op FT4 (pmol/L)	11.93 ± 1.54	24
Pre-Op thyroxine dose (mcg)	69.79 ± 35.34	24
Pre-Op HbA1c (%)	6.36 ± 1.75	24
Post-Op HbA1c (%)	5.41 ± 0.51	24
Pre-Op Vit D (nmol/L)	57.10 ± 26.20	23
Post-Op Vit D (nmol/L)	66.89 ± 23.68	23
Age (years)	40.08 ± 12.96	24

Pre-Op: Pre-operative; Post-Op: Post-operative; BMI: Body mass index; TSH: Thyroid-stimulating hormone; FT4: Free thyroxine; HbA1c: Hemoglobin A1C; Vit-D: Vitamin D.
